# Onion (*Allium cepa* L.) peel extract has anti-platelet effects in rat platelets

**DOI:** 10.1186/s40064-015-0786-0

**Published:** 2015-01-13

**Authors:** Ju-Ye Ro, Jin-Hyeob Ryu, Hwa-Jin Park, Hyun-Jeong Cho

**Affiliations:** Department of Biomedical Laboratory Science, College of Medical Science, Konyang University, 685, Gasuwon-dong, Seo-gu, Daejeon 302-718 Korea; Department of Biomedical Laboratory Science, College of Biomedical Science and Engineering and Regional Research Center, Inje University, 607, Obang-dong, Gimhae, Gyungnam 621-749 Korea; Present address: Department of Microbiology and Immunology, Institute of Medical Science, Tokyo University, Minato-ku, Tokyo, 108-8639 Japan

**Keywords:** Onion peel extract, Quercetin, Platelet aggregation, Intracellular Ca^2+^, Cyclic adenosine monophosphate, Thromboxane A_2_

## Abstract

The effects of onion peel extract (OPE) in collagen (5 μg/mL)-stimulated washed rat platelet aggregation were investigated. OPE inhibited platelet aggregation *via* inhibition of aggregation-inducing molecules, intracellular Ca^2+^ and thromboxane A_2_ (TXA_2_) by blocking cyclooxygenase-1 (COX-1) and TXA_2_ synthase (TXAS) activities in a dose-dependent manner. In addition, OPE elevated the formation of cyclic adenosine monophosphate (cAMP), aggregation-inhibiting molecule, but not cyclic guanosine monophosphate (cGMP). High performance liquid chromatography (HPLC) analysis of OPE revealed that OPE contains quercetin, one of the major flavonoids, which has anti-platelet effect. In conclusion, we suggest that OPE is an effective inhibitor of collagen-stimulated platelet aggregation *in vitro*. Therefore, it can be a promising and safe strategy for anti-cardiovascular diseases.

## Introduction

When normal blood vessels are damaged, platelets are activated by stimuli which are present in the walls of blood vessels, and induce aggregation (Nieswandt and Watson 2003). Platelets are activated by agonists such as collagen, thrombin and ADP, and it induces the signals by activating multiple G protein–mediated pathways to activate platelet-shape change, degranulation and aggregation (Nieswandt and Watson 2003; Offermanns 2006). But in many cardiovascular diseases such as acute coronary syndromes, atherosclerosis, stroke and peripheral vascular diseases, excessive platelet activation is regarded as the cause of thrombosis (FitzGerald et al. 1987; Davi and Patrono 2007; Kapoor 2008). Abnormal platelet aggregation leads to excessive TXA_2_ formation interacts with other platelets, inducing thrombotic disorders (Miller et al. 1977; FitzGerald et al. 1987). Therefore, the development of an anti-platelet agent could be a fundamental therapeutic approach to cardiovascular diseases (Bhatt and Topol 2003; Jackson and Schoenwaelder 2003). The activation of phospholipase C (PLC) through G protein, which results in the formation of inositol 1,4,5-triphosphate (IP_3_) and diacylglycerol (DG) plays an important role in platelet aggregation, contributing to elevated cytosolic free Ca^2+^ ([Ca^2+^]_i_) (Brass and Joseph 1985; Williamson et al. 1985). DG is hydrolyzed by DG lipase to produce arachidonic acid (20:4). The metabolic pathways of 20:4 lead to TXA_2_ formation which is one of the positive-feedback mediators during platelet aggregation through COX-1 and TXAS pathways (Baumgartner and Haudenschild 1972; Hamberg et al. 1975). On the other hand, cAMP and cGMP are known as anti-platelet aggregatory regulators. The major inhibitors of platelet activation are nitric oxide (NO) and prostacyclin (PGI_2_), which raise the levels of cAMP and cGMP (Trovati et al. 1997). The aggregatory effects of cAMP and cGMP on platelets are mediated *via* cAMP and cGMP-dependent protein kinases, which phosphorylate substrate protein, vasodilator-stimulated phosphoprotein (VASP) entailed in the inhibitory effect of platelet aggregation (Halbrugge et al. 1990). Thus, cAMP and cGMP are anti-platelet second messengers in platelet aggregation, and a substance which elevates the levels of cAMP and cGMP may control platelet aggregation.

Onion (*Allium cepa L*.) has been reported to have beneficial effects, including preventing stroke, coronary thrombosis, atherosclerosis, hyperlipidemia and hypertension (Bordia et al. 1975; Kawamoto et al. 2004). Especially, it has been reported to inhibit platelet aggregation induced by various agonists *in vitro* and *in vivo* (Moon et al. 2000; Bordia et al. 1996). In dogs, onion juice reduced collagen-induced whole-blood platelet aggregation (Briggs et al. 2001). Also, in rats treated with aqueous extracts of garlic and onion (500 mg/kg of body weight) for 4 weeks, TXB_2_ levels were significantly inhibited compared with that of control in serum (Bordia et al. 1996). These results may be linked by quercetin known as one of the most abundant flavonoids in vegetables (Crozier et al. 1997; Ewald et al. 1999). Epidemiological data suggest that those who consume a diet rich in quercetin-containing foods may have a reduced risk of cardiovascular diseases (Glässer et al. 2002; Kris-Etherton et al. 2004). Indeed, collagen-stimulated platelet aggregation was inhibited after ingestion of onion soup high in quercetin in a time-dependent manner (Hubbard et al. 2006). Therefore, *in vitro* and *in vivo*, onion has been concerned with anti-thrombotic effects by inhibiting platelet aggregation and activation (Kawakishi and Morimitsu 1988; Ali et al. 1999; Moon et al. 2000; Briggs et al. 2001). However, while those studies have investigated the effects of anti-platelet aggregation of onion bulb extract, the signaling for anti-platelet effects of the outer skin extract of onion have been barely analyzed. Therefore, this work was carried out in an effort to investigate the anti-platelet effects of outer skin of onion. In the present study, we investigated that OPE has inhibitory effects on platelet-mediated thrombotic disorders *via* down-regulation of TXA_2_ through reducing the [Ca^2+^]_i_, COX-1 and TXAS activities, as well as also up-regulation of cAMP levels in collagen-stimulated rat platelet aggregation without any toxicity *in vitro*.

## Results and Discussion

### OPE possesses quercetin in highest level

**Figure 1:**
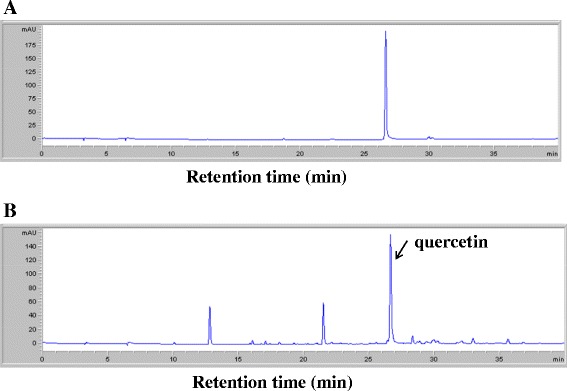
**HPLC chromatograms of OPE and internal standard (quercetin).** **(A)** The standard chromatogram of quercetin. **(B)** The chromatogram of OPE (0.5 mg/mL).

We analyzed quercetin of OPE with HPLC. In the previous study, quercetin has been reported to inhibit collagen-induced platelet aggregation (Hubbard et al. 2006). Also, it is known that outer skin of onion contains much higher levels of quercetin than its bulb (Hertog et al. 1992; Prakash et al. 2007). Therefore, it was used as a standard in this study. In Figure 1, HPLC analysis revealed that the retention time of quercetin was 26.6 min (Figure 1A) and OPE has three peaks mainly, the highest one proved quercetin (26.6 min) (Figure 1B). Calibration curve were linear over the range of 20 to 500 μg/mL of quercetin, and it was calculated from calibration curve. The content of quercetin from OPE was 167.3 ± 0.48 mg/g (16.7 ± 0.1% of quercetin in OPE) (data not shown). In addition, we examined platelet aggregation reactions in the presence of quercetin. The half-maximal inhibitory concentration (IC_50_) of quercetin in collagen (5 μg/mL)-induced platelet aggregation was 6 μg/mL (32.0 ± 4.0%, n = 3, data not shown). These results show that anti-platelet effects of OPE is linked with quercetin as a main component, although other two peaks were not unconfirmed (Figure 1).

### OPE inhibits platelet aggregation *via* down-regulation of [Ca^2+^]_i_ levels

**Figure 2:**
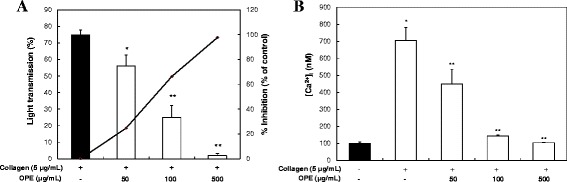
**Effects of OPE on collagen-induced platelet aggregation and [Ca** ^**2+**^ **]**_**i**_ **mobilization.** **(A)** Effects of OPE on collagen-induced platelet aggregation. Data are expressed as mean ± SD (n = 7). *p < 0.05 compared with that of collagen only. **P < 0.001 compared with that of collagen-induced platelet aggregation. **(B)** Effects of OPE on [Ca^2+^]_i_ mobilization. Data are expressed as mean ± SD (n = 3). *p < 0.05 compared with basal level. **P < 0.05 compared with that of collagen-induced [Ca^2+^]_i_.

Anti-platelet aggregation effect of OPE was determined. Washed platelets (10^8^ cells/mL) were activated with collagen (5 μg/mL) in the presence of 2 mM CaCl_2_ with or without various concentrations of OPE. Platelet aggregation rate induced by collagen only was 74.9 ± 2.7%, but OPE (50, 100 and 500 μg/mL) significantly inhibited platelet aggregation in a dose-dependent manner (56.4 ± 6.7, 25.3 ± 7.3 and 2.0 ± 1.2%, respectively) (Figure 2A). The inhibition rate was increased significantly by OPE (25.3%, 66.7% and 97.3%, respectively). These results suggest that OPE has anti-platelet effects in a dose-dependent manner. IC_50_ value of OPE was 80.0 μg/mL.

Intracellular calcium ions level ([Ca^2+^]_i_) play a key role in regulation of platelet function on their migration and adhesion (Detwiler et al. 1978). An elevation of [Ca^2+^]_i_ activates platelet aggregation (Nishikawa et al. 1980). In the previous study, quercetin has been reported to inhibit collagen-induced platelet aggregation through inhibition of [Ca^2+^]_i_ and glycoprotein VI signaling pathway (Hubbard et al. 2003). Therefore, we investigated if OPE inhibits [Ca^2+^]_i_ under collagen exposure. When Fura 2-loaded platelets (10^8^ cells/mL) were stimulated by collagen (10 μg/mL), the level of [Ca^2+^]_i_ increased from 98.2 ± 10.3 to 704.3 ± 76.7 nM (Figure 2B). However, this was significantly reduced by various concentrations (50, 100 and 500 μg/mL) of OPE (450.1 ± 85.4, 143.1 ± 7.0 and 103.6 ± 2.9 nM, respectively) in a dose-dependent manner. These results suggest that inhibitory effects of OPE on collagen-stimulated platelet aggregation was due to lowering of the level of [Ca^2+^]_i_, one of the key factor for platelet activation.

### OPE decreases the production of TXA_2_

**Figure 3:**
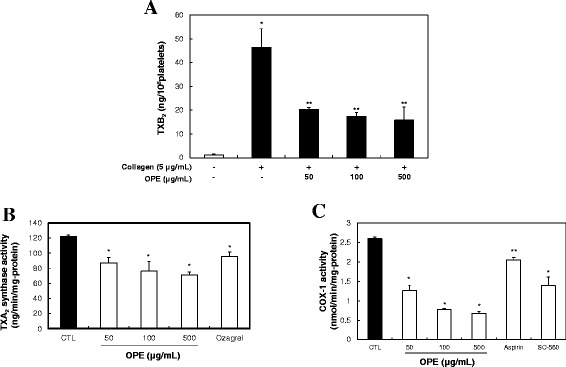
**Effects of OPE on TXA**_**2**_ **formation.** **(A)** TXA_2_ production by OPE. Data are expressed as mean ± SD (n = 3). *p < 0.05 compared with basal level. **p < 0.001 compared with that of collagen-induced platelets. **(B)** Effects of OPE on TXA_2_ synthase (TXAS) activity. Data represent mean ± SD (n = 3). *p < 0.05 compared with that of control. **(C)** Effects of OPE on COX-1 activity. Data represent mean ± SD (n = 3)*.* *p < 0.001 and **p < 0.05 compared with that of control.

TXA_2_ is a powerful stimulator and potent vasoconstrictor that is produced by platelets during their aggregation (Bunting et al. 1983; Cho et al. 2006). Collagen-stimulated aggregation of platelets induces αIIbβ3-mediated outside-in signaling and aggregation through the production of TXA_2_ (Cho et al. 2003). Also, aggregating platelets interact with coronary artery and TXA_2_ contribute to the direct activation of coronary smooth muscle by platelet aggregation (Houston et al. 1986). Therefore, TXA_2_ is considered as the important factor in thrombotic and cardiovascular diseases (Müller 1990). Therefore, we determined whether OPE reduce the production of TXA_2_ under collagen exposure. TXB_2_ (a stable metabolite of TXA_2_) levels in intact platelets was 1.2 ± 0.4 ng/10^8^ cells, and this was markedly increased to 46.4 ± 7.8 ng/10^8^ cells in the collagen-stimulated platelets (Figure 3A). However, various concentrations of OPE (50, 100 and 500 μg/mL) significantly reduced the production of TXB_2_ in a dose-dependent manner (20.4 ± 7.8, 17.3 ± 1.8 and 15.8 ± 5.5 ng/10^8^ cells, respectively). OPE strongly inhibited TXB_2_ level (inhibition rate: 65.9% at 500 μg/mL). In addition, quercetin (6 μg/mL) was inhibited TXB_2_ level from 37.2 ± 1.2 (control) to 25.2 ± 3.8 ng/10^8^ cells (32.3% of inhibition, n = 3, data not shown). These results show that the inhibitory effects of OPE on TXB_2_ production were linked with quercetin. OPE may be regulate platelet aggregation *via* down-regulation of TXA_2_ production which is one of the powerful stimulators of platelets activation. Based on these findings, we suggest that the consumption of OPE may prevent platelet-mediated cardiovascular disorders.

We next investigated whether TXB_2_ down-regulation by OPE is directly related to inhibition of its metabolic enzyme, COX-1 or TXAS activity.

### OPE inhibits TXAS and COX-1 activity

When platelets were stimulated by collagen, arachidonic acid (AA) converts to TXA_2_*via* COX-1 and TXAS activities (Hamberg et al. 1975). We determined whether OPE had the effect on TXAS or COX-1 activity. First, TXAS activity assay was assessed by measuring TXB_2_ formation. In Figure 3B, OPE (50, 100 and 500 μg/mL) significantly inhibited TXAS from 122.3 ± 1.5 (control) to 87.1 ± 7.2 (29% inhibition at 50 μg/mL) and 76.1 ± 12.6 (38% inhibition at 100 μg/mL) ng/min/mg-protein, respectively. 11 nM of ozagrel, a TXAS inhibitor, was used as a positive control and it decreased TXA_2_ level to 95.5 ± 5.9 ng/min/mg-protein (22% inhibition). These results indicate that OPE inhibited TXAS, one of the TXA_2_ metabolic enzymes, in a dose dependent manner.

We next assessed whether OPE affected COX-1 activity. After the addition of AA for 1 min at 37°C, COX-1 activity was determined by measuring fluorescent compound resorufin which the reaction between PGG_2_ and ADHP produced. In Figure 3C, OPE (50, 100 and 500 μg/mL) significantly inhibited COX-1 activity from 2.6 ± 0.1 (control) to 1.3 ± 0.2 (50% inhibition at 50 μg/mL) and 0.8 ± 0.1 (69% inhibition at 100 μg/mL) nmoL/min/mg-protein, respectively. 500 μM of aspirin and 330 nM of SC-560, COX-1 inhibitors, were used as positive controls. They inhibited COX-1 activities 2.1 ± 0.1 (19% inhibition) and 1.4 ± 0.3 nmoL/min/mg-protein (46% inhibition), respectively.

The inhibition of TXAS or COX-1 activity decreases TXA_2_ production and leads to anti-aggregatory effects (Gresele et al. 1991; FitzGerald et al. 1987). Therefore, the inhibitors of TXA_2_ production inducer (such as COX-1 and TXAS) can have the beneficial anti-thrombotic potential (Vilahur et al. 2007). In Figure 3, OPE reduced TXB_2_ production *via* regulation of COX-1 and TXAS activities. Therefore, our results suggest that OPE may have the beneficial property as an anti-platelet agent for cardiovascular diseases.

### OPE increases the formation of cAMP

**Figure 4:**
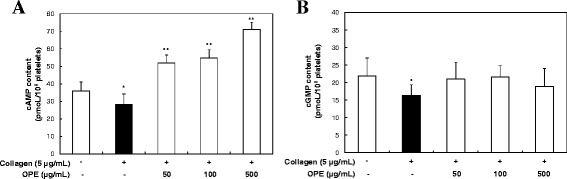
**Effects of OPE on cAMP and cGMP production in resting and collagen-stimulated platelets.** **(A)** Effects of OPE on cAMP production in resting and collagen-stimulated platelets. **(B)** Effects of OPE on cGMP production in resting and collagen-stimulated platelets. Data are expressed as mean ± SD (n = 3). *p < 0.05 compared with that of resting platelets. **p < 0.05 compared with that of collagen-stimulated platelets.

The elevation of the platelet aggregation induced by platelet stimulators is known to be lowered by either the production of cAMP or cGMP (Jang et al. 2002). We investigated whether OPE up-regulated the cellular level of cAMP or cGMP. In Figure 4A, collagen decreased intracellular cAMP level from 36 ± 5.1 pmoL/10^8^ cells (basal level) to 28.3 ± 6.0 pmoL/10^8^ platelets (control). However, when the platelets were incubated in the presence of both OPE and collagen (5 μg/mL), OPE (50, 100 and 500 μg/mL) significantly increased the cAMP level in a dose-dependent manner (51.8 ± 4.8, 54.7 ± 4.8 and 71.1 ± 4.1 pmoL/10^8^ platelets, respectively). On the other hand, OPE did not elevate the cGMP level (Figure 4B). These results indicate that OPE regulates the production of cAMP in collagen-stimulated platelets.

In platelets, it is known that cAMP- and cGMP-dependent effects inhibit agonist-induced increases in cytosolic calcium concentration and granule secretion (Aszodi et al. 1999). Considering these results, the inhibitory effect of OPE on [Ca^2+^]_i_ mobilization (Figure 2B) may be associated with up-regulation of cAMP levels by OPE (Figure 4A). Because the increased cAMP and cGMP levels affect activating PKA and PKG respectively, these enzymes phosphorylate their substrate proteins resulting in negative regulation of platelet aggregation (Li et al. 2003). Therefore, increased cAMP levels by OPE on collagen-induced platelet aggregation might be associate with the activity of PKA, and additional research in this area is recommended.

### OPE does not exert toxicity on platelets

**Figure 5:**
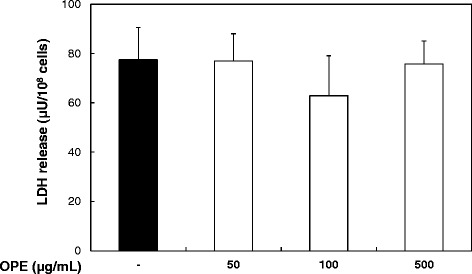
**Effect of OPE on the LDH release in washed platelets.** Data are expressed as mean ± SD (n = 3).

To determine whether OPE has potential toxicity, LDH release assay was examined. Washed platelets (10^8^ cells/mL) were incubated with various concentrations of OPE (50, 100 and 500 μg/mL) for 5 min as the same method of platelet aggregation reaction. Signs of toxicity were not observed significantly when platelets with OPE (50, 100 and 500 μg/mL) were compared to that of control (Figure 5). Therefore, the anti-platelet effects of OPE used in this study did not be affected by its toxic effect.

## Conclusion

We suggest that the inhibitory effects of OPE on collagen-induced platelet aggregation might involve the following pathway; OPE inhibits collagen-stimulated platelet aggregation without any toxicity *in vitro*, and has anti-platelet effects *via* up-regulation of cAMP levels but not cGMP and down-regulation of TXA_2_ through reducing the [Ca^2+^]_i_, COX-1 and TXAS activities. Therefore, OPE could be a promising strategy in many cardiovascular diseases such as atherosclerosis, myocardial infarction, and thrombosis.

## Methods

## Materials

Collagen was obtained from Chrono-Log Corp. (Havertown, PA) and LDH cytotoxicity assay, COX-1 fluorescent assay, and TXB_2_ EIA kits were bought from Cayman Chemical (Ann Arbor, MI). Fura 2-AM, ozagrel, acetylsalicylic acid (ASA), DMSO and quercetin were obtained from Sigma Chemical Corp. (St. Louis, MO). cAMP- and cGMP-EIA kits were purchased from BioVision, Inc. (Milpitas, CA). All other chemicals and reagents used in this study were obtained from Sigma Chemical Corp. (St. Louis, MO).

### Preparation of onion peel extract (OPE)

Onions were purchased from a local store in South Korea. 3 g of onion peels were washed in tap water twice and dried. Chopped onion peels were extracted with 60 mL of 70% ethanol at 100°C for 5 hr, and blended by vortex mixer for 10 min. OPE was centrifuged at 1,800 × g for 10 min and the clear supernatant was collected. The sediment was blended by vortex mixer for 10 min after adding 10 mL of D.W. The mixture was centrifuged at 1,800 × g for 10 min and the clear supernatant was collected. The above procedures were repeated for 5 times. Collected OPE was freeze-dried with a freeze dryer model (Ilshin BioBase Co., Ltd., Gyeonggi-do, South Korea) and re-suspended with D.W. to desired concentrations.

### Analysis of quercetin in OPE with HPLC

OPE dissolved in 50% methanol, and then it was analyzed by HPLC. An Agilent 1100 liquid chromatography system (Agilent Technologies, Santa Clara, CA), equipped with vacuum degasser, quaternary gradient pump, autosampler and diode array detector (DAD) connected to an Agilent ChemStation software. A Zorbax octadecylsilane (ODS) C18 column (250 mm × 4.6 mm id, 5 μm) and a Zorbax ODS C18 guard column (12.5 mm × 4.6 mm id, 5 μm) were used at a column temperature of 25°C. The mobile phase consisted of water with 0.1% acetic acid (A) and acetonitrile (ACN) (B) using the following program: 0–30 min, 0–50% B; 30–40 min, 50–100% B. The flow rate was at 1.0 mL/min and sample injection volume was 10 μL. The UV detection was operated at 254 nm. Three concentrations of standard quercetin were injected in duplicate, and then the calibration curve was constructed by plotting the peak area against the concentration of analyte.

### Preparation of washed rat platelets

Rats (8 week-old, male, Sprague–Dawley, specific pathogen-free) were purchased from Taconic Farm., Inc. (Hudson, NY). Rats were housed with a 12 hr light–dark cycle, and fed a normal diet. Tap water was freely available. The rats were sacrificed after starvation for 12 hr. They were anesthetized with ethyl ether, and blood was collected from the abdominal vein with 3.2% sodium citrate (1:9, v/v). Platelet-rich plasma (PRP) was centrifuged at 125 × g for 10 min to remove the red blood cells and PRP was separated with platelet-poor plasma (PPP) and platelet layer. Platelets were washed twice with washing buffer (138 mM NaCl, 2.7 mM KCl, 12 mM NaHCO_3_, 0.36 mM NaH_2_PO_4_, 5.5 mM glucose, and 1 mM EDTA, pH 6.9). The washed platelets were resuspended in suspension buffer (138 mM NaCl, 2.7 mM KCl, 12 mM NaHCO_3_, 0.36 mM NaH_2_PO_4_, 0.49 mM MgCl_2_, 5.5 mM glucose, pH 7.4) to a final concentration of 5 × 10^8^ platelets/mL. All of the above procedures were carried out at 25°C to avoid platelet aggregation on cooling. All animal experiments were carried out according to the guidelines of Konyang University (Daejeon, South Korea) and Ethics Committee.

### Measurement of platelet aggregation

Washed platelets (10^8^ cells/mL) were pre-incubated for 3 min at 37°C in the presence of 2 mM exogenous CaCl_2_ with or without OPE (50, 100 and 500 μg/mL). After the incubation, washed platelets stimulated with collagen (5 μg/mL) for 5 min. Aggregation was monitored using a Chrono-Log aggregometer at a constant stirring speed of 1,200 rpm (Breddin 2005; Born and Carlo 2006; Maione et al. 2011, 2014). Each aggregation rate was evaluated as an increase in light transmission. The suspension buffer was used as the reference. Quercetin was dissolved in 0.5% of DMSO, and the effect of DMSO was subtracted from the results.

### Determination of [Ca^2+^]_i_

PRP was incubated with 5 μM Fura 2-AM at 37°C for 60 min. Because Fura 2-AM is light-sensitive, the tube containing the PRP was covered with aluminum foil during loading. Fura 2-loaded washed platelets (10^8^ cells/mL) were pre-incubated for 3 min at 37°C with various concentrations of OPE (50, 100 and 500 μg/mL) in the presence of 2 mM CaCl_2_ and then stimulated with collagen (10 μg/mL) for 5 min for evaluation of [Ca^2+^]_i_. Fura 2 fluorescence was measured with a RF-5301 spectrofluorometer (Shimadzu Corp., Kyoto, Japan) with an excitation wavelength that changed every 0.5 sec from 340 to 380 nm; the emission wavelength was set at 510 nm. The [Ca^2+^]_i_ values were calculated using the method of Schaeffer (Schaeffer and Blaustein 1989).

### Measurement of TXB_2_

Washed platelets (10^8^ cells/mL) were pre-incubated with or without OPE (50, 100 and 500 μg/mL) for 3 min in the presence of 2 mM CaCl_2_, and activated for 5 min with collagen (5 μg/mL) for 37°C. The reactions were terminated by the addition of ice-cold EDTA (5 mM) and indomethacin (0.2 mM). The amount of TXB_2_, a stable metabolite of TXA_2_, was determined using a TXB_2_ EIA kit according to the manufacturer’s recommendations. Quercetin was dissolved in 0.5% of DMSO, and the effect of DMSO was subtracted from the results.

### TXAS activity assay

Platelets in a suspension buffer containing 1% protease inhibitor were sonicated. The platelet lysates (10 μg-protein) were pre-incubated with or without OPE (50, 100 and 500 μg/mL) at 37°C for 5 min. The reactions were initiated by the addition of PGH_2_. After incubation for 1 min at 37°C, the reaction was terminated by the addition of 1 M citric acid and neutralization with 1 N NaOH. Ozagrel (11 nM) was used as a positive control of TXAS inhibitor. The amount of TXB_2_, a stable metabolite of TXA_2_, was determined using a TXB_2_ EIA kit according to the manufacturer’s recommendations.

### COX-1 activity assay

Platelets in a suspension buffer containing 1% protease inhibitor were sonicated. The platelet lysates (10 μg-protein) were incubated with or without various concentrations of OPE (50, 100 and 500 μg/mL) for 30 min at 37°C. The reactions were initiated by the addition of arachidonic acid. After incubation for 1 min at 37°C, COX-1 activity was assayed with COX-1 activity fluorescent assay kit according to the manufacturer’s recommendations. SC-560 (330 nM) and ASA (500 μM) were used as a positive control of COX-1 inhibitor. COX-1 activity was measured with Synergy HT multi-model microplate reader (BioTek Instruments, Winooski, VT).

### Measurement of cAMP and cGMP

Washed platelets (10^8^ cells/mL) were pre-incubated for 3 min at 37°C with various concentrations of OPE (50, 100 and 500 μg/mL) in the presence of 2 mM CaCl_2_, and then stimulated with collagen (5 μg/mL) for 5 min for platelet aggregation. The aggregation was terminated by the addition of 80% ice-cold ethanol. cAMP and cGMP were measured using cAMP and cGMP EIA kits according to the manufacturer’s recommendations.

### LDH assay

To assess whether OPE has toxicity, we examined the effect of OPE on LDH release *in vitro*, which is a stable enzyme normally found in the cytosol of cells, but rapidly releases into the supernatant upon damage of cell membrane. Washed platelets (10^8^ platelets/mL) were incubated for 5 min at 37°C with various concentrations of OPE (50, 100 and 500 μg/mL), and then the supernatant was measured by an LDH assay kit according to the manufacturer’s recommendations.

### Statistical analysis

The experiment results are expressed as the mean ± SD Statistical analysis was performed by two tailed unpaired Student’s t-test or ANOVA as appropriate. If this analysis indicated significant differences among the group means, each group was compared by the Scheffe’s method for post hoc tests (Brown 2005).
